# The New York Institute of Stomatology

**Published:** 1901-06

**Authors:** 

**Affiliations:** The New York Institute of Stomatology


### Reports of Society Meetings.

### THE NEW YORK INSTITUTE OF STOMATOLOGY.

A regular meeting of the Institute was held at the office of Dr. E. S. Robinson, No. 28 West Thirty-ninth Street, New York, on March 5, 1901, the President, Dr. J. Morgan Howe, in the chair.

The minutes of the last meeting were read and approved.

### COMMUNICATIONS ON THEORY AND PRACTICE.

*Hypertrophy of Upper and Lower Jaws.*

*Dr. S. E. Davenport.*—Dr. Kasson C. Gibson regrets his inability to be present this evening, and has very kindly sent for our examination a model representing a case of extreme hypertrophy of both upper and lower jaws, more particularly of the gum tissue. The patient is a man twenty-nine years of age and in general good health in other respects. He was referred to Dr. Gibson by Dr. Bull, the surgeon. The patient has, I understand, recently been operated upon and the hypertrophied tissue removed. Dr. Gibson has been kind enough to promise that when he has an opportunity he will take a model of the mouth representing the condition after the operation, which he will present to the society.

*Inlay Work.*

*Dr. C. F. Allan.*—I do not know that there is anything new I can present regarding the subject in which I am so much interested, inlay work, but I have thought that possibly in overcoming some of the difficulties which I have met, especially in approximal work, I may have gained experience which will be of use to other dentists.

In making matrices probably more difficulties are to be encountered than with any other part of the work. Where the cavities are not on approximal surfaces I have been in the habit of putting my piece of No. 30 gold, of which my matrix is to be made, on a sheet of spunk, and then with an apple-seed burnisher pressing the gold into the spunk, to in some measure approximate the size and shape of the cavity; then, when the gold is placed in the cavity and pressed in carefully with spunk, a clean matrix will generally be made without any break in the gold. Most frequently, however, in approximal cavities with teeth close together this procedure is impossible, and with cavities moderately deep it is often impossible to get a matrix that is not seriously broken at the bottom of the cavity, sometimes, indeed, with the bottom broken almost entirely away but with all the margins perfect. In such cases I find that by using the smallest scrap of No. 3 foil, a scrap not much larger than the bottom of the matrix, putting it on the investment and then placing the broken matrix over this scrap of gold, by the gentle tapping necessary to settle the matrix I have a bottom and a perfect mould. Another difficulty is in cutting the little notches in the sides of the inlay. The smaller inlays are very difficult to handle with the fingers, and with the extremest care the operator is in constant danger of losing them. If one will, however, keep in his cabinet half a dozen or so orange-wood sticks tipped with shellac, all he has to do is to hold the point of one of them over his Bunsen burner and it is but a matter of a few seconds when he has his inlay held in a practical way that admits of no losing and makes easy this otherwise troublesome detail. Another difficulty with me was in connection with the investment. Asbestos powder moistened either with alcohol or water allows the matrix to drag, and this unsettled and easily moved condition of the matrix was very annoying, but by using fyrite, a new investment material sold by the S. S. White Company, this difficulty was overcome. The matrix holds solidly in it and it makes a perfect investment material for the low-fusing bodies where gold is used for the matrix, but it should not be used for the high-fusing bodies. Possibly it may need a little more careful drying, taking a minute or more time, but otherwise it is all right.

*The President.*—We have present with us this evening Dr. Eugene Smith, of Boston, and Dr. Brackett, of Newport, also Dr. Fillebrown, Dr. William P. Cook, Dr. Taylor, and Dr. Bradley. We are especially favored in having all these gentlemen present with us, and we hope to hear from all of them before the evening is over.

I have the pleasure, gentlemen, of presenting to you Dr. George A. Maxfield, of Holyoke, who will read a paper on "Malignant Growths in the Mouth, Incidental to General Practice."

(For Dr. Maxfield's paper, see page 373.)

DISCUSSION.

*The President.*—In the discussion of this interesting paper we are to have the pleasure of listening to Drs. Dawbarn and Coley, after which we hope to have the pleasure of listening to others present who may wish to join in the discussion. Dr. Dawbarn, gentlemen.

*Dr. R. H. M. Dawbarn.*—Mr. President and gentlemen, the chairman of the Executive Committee has twice done me the honor to ask me to be very full in the discussion of this paper. If I were to take him at his word I am afraid that the discussion would occupy all of this evening and some of to-morrow. It will only be possible for me to touch here and there. I would rather leave intervals which, if questions are asked afterwards, I may be permitted to then fill in. I will discuss the subject of diagnosis and treatment in the following order, taking up the malignant conditions of the soft parts first, afterwards those of the hard parts. In the interesting paper that has been presented to us this evening the morbid condition known as "leucoplakia" has been mentioned. As the name implies, it is of a whitish appearance, sometimes called "wash-leather," and may involve any surface within the mouth,—lingualis, buccalis, or gingivalis. This wash-leather appearance, in about twenty-five per cent. of cases, is the precursor of epithelioma. This disease occurs very much more frequently in those addicted to excessive smoking. Avoid everything irritating in treating it, such as the use of nitrate of silver or any other caustic. Stop smoking. Use only soothing mouth-washes. None but radical operations should be performed for its cure; and whether to operate will depend upon the differing conditions in each case. As to cancer of the tongue:

*Diagnosis.*—To be distinguished chiefly from syphilis of the tongue. There are quite a number of distinguishing features,—viz., cancer of the tongue (epithelioma) is almost the only type of malignant growth of the tongue; it begins almost always from a decayed tooth as an irritant factor. Although not the actual cause of cancer, mechanical irritation is generally the predisposing cause. This is again shown in cancer of the scrotum as found in chimney-sweeps. Indications of syphilis are more frequently on the dorsum of the tongue; cancer, as stated, begins at the point of tooth irritation. Cancer generally presents a single ulcer, while syphilis is often seen in more than one place on the tongue. Cancer is the more painful of the two; it occurs in patients a great deal older, over forty years, as a rule, while syphilis occurs more frequently under that age. Cancer is oftener found in the male, syphilis more frequently in the female. The discharge is more offensive in cancer. In cancer it is only the neighboring lymphatic glands beneath the lower jaw that are involved, while in syphilis the enlarged lymph-nodes are more diffused, occurring elsewhere in the body quite as much as near at hand. But after all, the diagnosis upon which we are chiefly to rely is what is revealed to us by the microscope. In the case of a cancer, upon removing a small piece you will find nests of epithelial cells surrounded by a stroma. In the case of syphilis we find small round-celled infiltration. Indeed, syphilis has been called a "disease of the degraded leucocyte."

Another ulcer occasionally seen here is that of tubercular diseases. Although this ulcer may somewhat resemble the others, it is excessively rare for it to occur as a primary condition, and generally there is evidence of very advanced tuberculosis elsewhere. The microscope will reveal the tubercle bacilli, etc., in a section removed for examination. We can also have ulcers due to simple uncleanliness, of course, the microbes of the mouth, of which there are some twenty varieties in the human saliva, setting up an irritation in certain cases. Some of these microbes are pretty virulent. The microscope will also help us here. So much for differential diagnosis.

*Treatment.*—In carcinoma of the tongue I hardly need say that there is only one treatment, which is prompt and radical surgery. Every week, or certainly every month, I see cases in my hospital and elsewhere that have gone too far for hopeful results. Perhaps one reason for this is, that on account of the surgeon suggesting the removal of the whole of the tongue, the patient delays the operation until it is too late. Inasmuch as these growths generally occur at the extreme lateral margin of the tongue, the operation for the removal of one-half the tongue will be just as effective, in most instances, as the more radical operation of the removal of the entire tongue, and with half a tongue the patient can speak fairly well. In so far as blood-supply and lymphatics are concerned, we have practically two tongues. It is easily shown, by injecting one of the lingual arteries of a cadaver with blue solution, that one-half, exactly, of the tongue will be blue, and the other will remain red, there being no anastomosis between the two halves except sometimes at the very tip.

There are a number of interesting points in connection with excision of the tongue, or part of it. As ordinarily done, in my estimation, three or four mistakes are made. I think the Whitehead operation should never be done. This operation is made entirely within the mouth. It is akin to performing an operation for cancer of the breast without cleaning out the armpit glands. The lymph-nodes (glands) beneath the jaw should always be removed, because they will frequently develop cancer later on, being really infected, though not to be felt at first. For this reason the tongue should be removed from beneath the jaw, as then the glands can at the same incision be attacked. In this incision there is the great advantage, too, that we can easily expose and tie the lingual artery before cutting the tongue, and thus the operation is rendered almost a bloodless one. If it is possible to do so, without approaching the cancer, I think it wise to save the last two or three centimetres of the tongue, instead of removing it entirely down to the hyoid bone; this to aid the epiglottis in its work. We should save the twelfth nerve, which is entirely a motor nerve, and which can readily be attached to the raw surface of the stump of the tongue, thus innervating it. This will enable the epiglottis the better to be brought down on the top of the larynx in swallowing. I think this is a new point and a good one; it is one I have employed. It is extremely important, where the tongue is removed far enough back to affect swallowing, that for a long time the patient be kept in an inverted position,—that is, with the head a little lower than the chest,—so that the rank and fetid saliva and discharges shall not gravitate into the larynx and so on towards the lungs. The patient after this operation is especially threatened with a septic pneumonia from this cause. He can be kept for weeks in this position if need be, and should not be allowed to sit up until the parts are well granulating and the saliva is quite odorless again, or, better, learned to close the epiglottis.

In those cases of cancer of the tongue which have gone beyond the reach of radical cure there are still certain things that may be done. I do not know of anything in surgery quite as near like magic in its dramatic results as the outcome of the division of the lingual nerve (the so-called gustatory nerve), where there is intense suffering in cancer of the tongue and where it has gone beyond the use of the knife. The pain sometimes is so great as to bring tears to the patient's eyes at every act of deglutition or speech. The lingual nerve, which is the one causing the suffering, can be divided in five seconds' time under cocaine, by introducing into the mouth a curved bistoury two centimetres below and behind the grinding surface of the last molar tooth and cutting against the ramus of the jaw towards that tooth. There is nothing else of any importance that can be injured here, and the instant the section is made the patient cannot feel any pain in this half of the tongue, in front of the circumvallate papillæ. Of course, the nerve will grow together again in a shorter or longer time, but it can be as easily divided again.

### *Bony Malignant Growths.*

Of these, the ones most frequently seen by dentists are those of epulis springing from the socket of a tooth. They may or may not be sarcomatous. In any case one should not hesitate long. Many tumors of the mouth and elsewhere, although benign in their incipiency, will frequently develop malignant characteristics in time. If a fungous growth that has been curretted away returns, no attempt should be made to retard it by means of pressure, as by a gutta-percha plug, as has been done, nor with leucoplakia, should it be treated with irritants. It is a case for a surgeon; and the treatment should be either nothing or absolutely radical. It is best not only to currette away the growth, but a considerable portion of the alveolus in that region should be removed by the gouge. Early diagnosis of bony malignant growths about the jaw and elsewhere has brought out one point in my experience which I have repeatedly alluded to before in this connection. Every one knows that not infrequently both sarcoma and carcinoma decalcify bone, and that fact can be used in early diagnosis. Our honored president remembers a case which he referred to me in 1896, in which a dentist extracted one of the bicuspids in the upper jaw for a severe face-ache. He noticed with casual interest that the tip of the root, which should have been of stony hardness, was soft. That ought to have been to him a striking evidence of a malignant growth. However, the dentist did not think of it, and subsequently the case came to Dr. Howe. He suspected a malignant condition, and sent the case to me. I have here a specimen, which some of you have seen, and which indicates the condition. (Upper jaw removed from this patient was here shown with sarcoma filling the antrum.) I made the diagnosis by taking an ordinary sewing needle, and I found that I could pass this needle into the bony roof of the mouth with about the same resistance as in ordinary cartilage. In a good many instances this will be of help to you. If you can run the needle into the bone, or even engage the point in bony tissue, which should be dense, a malignant growth should be suspected.

*Prognosis.*—I starved the region by cutting out the external carotid on both sides. And this brings up a very interesting question of treatment. When one is able to excise a cancer and cut well beyond its limits, it is of course the wise thing to do. When this is impossible, and unless the antitoxin treatment succeeds (and the usefulness of the latter seems limited to one type of sarcoma alone), then the patient is going to die within a few months with absolute certainty. There is nothing more hopeless than cancer of the mouth or jaws which have got beyond the possibility of excision. There is but one hope left in these otherwise hopeless cases, and that is the starving of the growth by cutting off its blood-supply. By that I do not mean simply *tying* both of the external carotids, but cutting them out from end to end, tying off and dividing all the branches in the process. Ligating the internal or the common carotid would cut off the blood-supply from the brain. It is simply desired to cut off the blood-supply from the superficial parts.

It seems proved that the normal tissues can get along with very little blood as compared with that required by a malignant growth, and when the blood-supply is cut off from this growth, although it does not disappear, it shrinks, and in most instances does not resume its growth. In sarcoma I have been particularly successful, less so in carcinoma. I will say this, that I have seen many cases, otherwise utterly hopeless, which by this method have had many months of life. Although I would not promise a cure, I can at least promise this. The method consists in cutting off the blood-supply from the external carotids on both sides. As I have said, it consists in not merely tying these arteries, for if only that were done the pulse would return inside of a week so that it could be felt in the temporals and facials. There are thirty or forty ways in which by anastomosis this might happen. It first occurred to me six years ago, and I then tried it at once, that a more radical way might be carried out by cutting out the entire length of both of the external carotid arteries. This sounds like a pretty dangerous operation, but it is not excessively difficult to do. It can be done inside of half an hour. I never attempt to do it on both sides at one operation, however. The only case I know of where this was attempted resulted in the death of the patient from shock, at the hands of another operator. I prefer to do it in two operations at intervals of two weeks. I have now done this operation in over forty instances. In several cases, after the operation on the affected side first, there was so much shrinkage of the growth that the patient, mistakenly thinking himself cured, refused to have it done on the other side. Now I always do it on the sound side first, and then the patient, finding there is no change, will consent to having it done on the other side. The operation has also been done by Dr. Keen and Dr. DaCosta, of Philadelphia, and in New York by Drs. Blake, Bremen, Weir, Lilienthal, W. Meyer, Collins, and others, with a very low rate of mortality. In every one of these instances the patient was cachectic, and from that fact one would expect a greater mortality. I would be willing to assert that in experienced hands the operation will not result in over five per cent. of deaths. The amount of blood lost need not exceed an ounce. You may ask how these parts can now get any blood-supply at all. There are two at least, one from the internal carotid, which by its infraorbital branch anastomoses with the angular from the facial; the other through the occipital branch of the external carotid; the princeps cervices branch of this artery anastomosing with the profunda cervicis coming from the first intercostal artery. It is perfectly feasible to cut off still further the blood-supply by tying the infraorbital and occipital if desired. It has been suggested by Dr. Wyeth, in order to still further cut off the blood-supply from the smaller branches, that the internal maxillary artery, for example, be injected with boiling water. Another suggestion of Dr. Wyeth is the injection of sterilized paraffin, or some combination of wax which at the temperature of the body would again become solid.

It is a question, however, whether this would not seriously threaten sloughing of the part; and, of course, we would not try such means until after carefully experimenting upon the lower animals. We are justified, however, in taking radical measures when we remember that these are hopeless cases, directed straight to the other world in a very short time unless the disease is checked by some means; and that medicines, and even ordinary surgical intervention, are wholly useless in the more advanced cases. Mr. President, I have already taken up more time than I meant to. I will therefore conclude, and if there are any questions, I shall be very glad to answer them to the best of my ability.

*Dr. Wm. B. Coley.*—The demonstration has already brought out most of the points that are of great interest. What I have to say will be based upon my own clinical experience in these cases of cancer of the jaw.

I have personally had twenty-five cases of cancer of the upper and lower jaw, eighteen of the upper and seven of the lower jaw. The patients ranged all the way from twenty-two to seventy-six years. The majority were comparatively elderly. Five cases were between sixty-five and seventy-six. Sarcoma was by far the most frequent. I have seen only one case of carcinoma of the upper and none of the lower jaw. The malignancy of these different growths varies very much. Growths in the lower jaw are more dangerous to life than those in the upper. I have some notes of cases of sarcoma of the lower jaw which I will mention.

1. Disease occurring in a woman aged forty-eight. Killed her in four months. The disease started, as most of these do, almost exactly as an inflammatory growth, apparently beginning in an alveolar abscess. There was a small amount of pus. Two or three different physicians saw her, and it was washed out by various solutions. The diagnosis was not made until the tumor had reached a large size. The question of pain is very important in distinguishing whether the trouble is sarcomatous or inflammatory. In the malignant growth the pain is seldom severe, while in the inflammatory process it is more marked. I doubt the wisdom of Dr. Maxfield's suggestion of using washes in these doubtful cases, thus losing several weeks of valuable time. In every doubtful case a small piece should be snipped out and submitted to a microscopical examination.

2. Woman thirty-nine years of age. Operated upon in Ohio ten years before. Operation done comparatively early. Half the lower jaw resected from the symphysis to the joint. Remained perfectly well for ten years, then there was an extensive local recurrence, as shown by the photograph. Treated her for several weeks with the mixed toxins but with no apparent benefit.

3. Another case of late recurrence of sarcoma of the lower jaw, operated upon five and a half years prior to the recurrence. Entire half of the jaw removed. Did not recur in the glands nor in the jaw, but in the mastoid portion behind the ear. Treated with mixed toxins for two or three months. At first there was considerable diminution in size, but after a while the toxins seemed to lose control, and I was obliged to abandon it.

4. Sarcoma of the nasal fossa, afterwards invading the antrum. Treated with antitoxins, and it was one of the few in which there was any marked improvement. The case was operated upon by Dr. Bull in the New York Hospital. It was a round-celled sarcoma, and recurred promptly after the operation. Under the antitoxin treatment the tumor almost entirely disappeared, and he was sent out of the hospital. Very soon, however, it recurred, and although the toxin treatment was begun again, it seemed to have no effect. He went on rapidly from bad to worse; the growth became very vascular, and in a very bad cachectic condition he was operated upon by Dr. Dawbarn. I believe he is one of the few cases of death by this operation.

The early histories of a few of these cases show what some of the early symptoms are.

1. Sarcoma of the lower jaw; age, thirty-seven. The first she noticed was pain or discomfort in the region of a tooth that had been drawn some time before. History of an injury, having struck that side of the face against a door. This question of injury Dr. Dawbarn has already referred to. In nearly all of my jaw cases there was a history either of injury from wearing an ill-fitting plate or from pulling a tooth. Several patients have stated that the tooth was drawn with great difficulty, and in doing so a piece of the jaw was broken. This generally antedates the tumor some weeks or months. It is a very common history.

2. Case of very great interest; a young man of twenty-four; treated by Dr. Spencer, of Watertown, N. Y. Growth soon reappeared. It was a spindle-celled sarcoma. He declined to have a radical operation. He was treated with toxin for about a year, when the growth almost disappeared. The granulations which came out of the lower opening of the antrum became less and less vascular, and finally almost altogether ceased to appear. The tumor then began to grow and did not respond to larger doses of the toxins. Impossible to control it. The patient died within a year and a half of the beginning of the disease.

3. One more case of great malignancy, first seen October 2, six years ago. The first symptom was a small ulceration in the alveolar process of the lower jaw. The patient was forty-eight years of age. Within five weeks the whole mouth was filled up. The glands in the neck were enlarged. He could not swallow anything. Died within three and a half months of the beginning of the disease.

Dr. Dawbarn has already spoken rather fully regarding the diagnosis of these tumors. I would like again to emphasize the point of early diagnosis. Regarding the question of pain, the general popular idea is that all malignant growths are very painful, and unless the patient complains of a great deal of pain the physician or dentist is not impressed with the condition. It is these painless swellings in the jaw and elsewhere which are so dangerous. From the very fact that they are not painful at first, and are not recognized perhaps until they become painful, it may be too late. I think, especially in the jaw where these growths are unusually malignant, any abnormality in the way of a growth or a thickening of the parts should not be neglected, but a portion be removed under cocaine and examined microscopically at the earliest possible moment.

The prognosis in the lower jaw is absolutely bad. In nineteen cases collected by Butten none died of operation, twelve died within a year, only a single case lived as long as two and a half years, and not a single one of the nineteen passed the three-year limit. The upper jaw is not quite so bad. Ten or fifteen years ago it was very much worse than now. The latest statistics of Martens, which are the only ones carefully traced, show that out of eighty-four cases operated upon, seventy-four being complete resection of the upper jaw, sixteen lived beyond three years. Of these cases, five were epithelioma, three carcinoma, two round-celled sarcoma, one spindle-celled sarcoma, one endothelioma, and one giant-celled sarcoma.

Regarding the toxin, I do not recommend it except in cases of sarcomatous growths. Dr. Dawbarn's statement was not quite right when he said it was applicable only to one kind of sarcoma. I have found it to work better in the spindle-celled variety, but it is applicable to all kinds of sarcoma. I have treated four cases of round-celled sarcoma with success; that is, they have lived more than three years. One of these was sarcoma of the upper lip. She was treated four years ago. It was certainly malignant. It has entirely disappeared, and the patient is in perfect health.

In a case of round-celled sarcoma of the mesentery, the patient has been well more than five years. In the spindle-celled, which is the most favorable variety for the antitoxin treatment, the prognosis is better. Of the cases which I have treated with this method, I have had fifteen which have remained well from three to eight and a half years. Up to 1898 there had been thirty-five successful cases treated by this method by other men, and ten of these were round-celled. When you consider that these cases were entirely hopeless, it must be admitted that something has been accomplished. I think it shows that the toxin has a place in the treatment of these diseases. Of course, it is a very great pity that it does not act as well on carcinoma and the various other forms of sarcoma as it does on spindle-celled. It seems to me that we have here a clue to the direction of the future treatment of malignant diseases. Supposing carcinoma and sarcoma to be due to some form of microparasite, we are not likely to get any great improvement in our present surgical results, and we cannot hope to successfully treat the disease until we know something of its cause. I think we shall know more about this in the next ten years. Large sums of money are being left every year to investigate this problem and some of the best minds in the profession are at work on it.

I thank you for the courteous invitation from the chairman of your Executive Committee, and have enjoyed being with you very much.

*Dr. C. A. Brackett.*—I would like to ask Dr. Coley for some explanation as to the nature of these toxins and how they are used.

*Dr. Coley.*—The first preparation used was a living culture of erysipelas. I started on this line in 1891, the result of an interesting case of sarcoma. It was the case of a patient who had been operated on four times by Dr. Bull. At the last operation he exposed the carotid artery, and, finding it impossible to remove the growth, he closed the wound and gave the patient up to die. The patient had an attack of accidental erysipelas, and two weeks afterwards a second severe attack. I found the patient alive and well seven years afterwards. This case led me to this line of investigation. The original preparation was a pure culture of the streptococcus of erysipelas. I used it in twelve cases, with two fatalities from erysipelas. In some cases I had great difficulty in producing erysipelas. In one case, although I injected the living culture, I could not produce the disease, but I was able to control the malignant growth. This led me to discard the pure living culture and take up the toxin, which could be used with perfect safety. This can be sterilized with a little thymol and kept for several months. It is prepared by Messrs. Parke, Davis & Co. I find the toxins vary greatly, and have attributed my ill success in many cases to this fact.

*Dr. Maxfield.*—I would like to ask Dr. Dawbarn if there is any liability of diagnosing actinomycosis as carcinoma or sarcoma?

*Dr. Dawbarn.*—I do not consider the diagnosis ordinarily to be difficult. Actinomycosis is caused by a ray-fungus, which can be seen and recognized very readily by a very low magnifying-glass. Still, it is admitted that when there are very few actinomycetes present, and the disease does not cause suppuration, but merely a slowly developing growth, that chronic form of actinomycosis may extremely closely resemble sarcoma. It may be almost impossible to differentiate.

*Dr. Brackett.*—I cannot attend a meeting of the Institute of Stomatology without at least expressing my gratification at being permitted to come, and I certainly have the liveliest appreciation of the instructive material that has been placed before us this evening. As a dental practitioner I have been greatly impressed with this subject, and with the importance of the discrimination which is necessary in dealing with these cases. I think, on the one hand, that it is very greatly to be desired that the mind of the patient should not be unnecessarily alarmed. There have been numerous instances of extreme apprehension being aroused by the inconsiderate and not always intelligent expressions of the dental practitioner. On the other hand, I think the dentist should be sufficiently conversant with the ordinary indications of malignancy and of benignancy to guide the patient in the safe direction. I have also been much impressed with the doctrine, so well expressed by Dr. Garretson, that when a pathological growth in the mouth is not of a nature explainable as any of the common pathological conditions, such as inflammation, abscess, hypertrophy, ranula, etc., are explainable, then the growth is to be treated with the latitude due to cancer. Every dentist has the opportunity of allaying in patients' minds groundless apprehensions concerning new growths, and doing this is fulfilling a most beneficent mission. However, whenever a growth appears at a time of life and under conditions which cause a suspicion of malignancy, that patient should at the earliest possible moment have the advantage of the best skill. All of us see these cases at times, and in all cases we should be alert to exercise our resources to set aside influences which may occasion malignant growths, or which may tend to change the character of growths which in their inception were benign. An operation, when it is undertaken, should be undertaken by a person of ability, and should be radical, reaching beyond the region of diseased tissue. One word with regard to the specimen presented, the diagnosis of which was made by sticking a needle into the bony structure. Such a growth may be impressively demonstrated by putting a small electric lamp in the mouth in a dark room. The difference of translucency of the two sides would be most marked.

*Dr. Dawbarn.*—Transillumination was used in this case. It is extremely essential in all cases of antrum trouble. The simple test of the needle, however, is also very useful in this connection.

*Dr. T. Fillebrown.*—Mr. President, I have experienced great pleasure in listening to the paper and the discussion. I shall attempt to add but little. The remark was made that pressure is useless in these cases. My experience indicates that it may sometimes be effective. I had a case of epulis which occurred between the lower bicuspids. The patient was a lady forty years of age, of an extremely nervous type. I think she would have died rather than have submitted to the knife. She would not think of any violence being done whatsoever. I took some cotton and, placing on it some strong persulphate of iron, pressed it in between the teeth. At the next visit the growth was very much shrunken away. I continued to do this, and within the course of two weeks it was completely cured and never returned again. Usually I would have used the knife or engine and gouged the growth away. Most patients would submit to it. If I had another patient of this same temperament I should resort to the same means. In another case of a lady over seventy years of age there appeared a cancer between the median line and the third molar tooth. It was decided to remove the growth, which I did, making a hole in the roof of the mouth three-fourths of an inch in diameter. I lengthened out her artificial plate to cover it. That was nearly three years ago, and the place has remained entirely well; indeed, it has almost filled with granulations. Bunches have, however, appeared around the tuberosity of the same side, so that her plate has of necessity been gradually cut away. As she is over seventy years of age, she will probably not submit to another operation. I would like to ask Dr. Coley if the toxins have ever been used to prevent a return of the disease.

*Dr. Coley.*—I have never used the toxins in primary operable growths, only in inoperable cases. I believe the toxins will be found to be very valuable, used as prophylactic after primary operations without waiting for recurrences. I would deprecate small operations. The operation should be radical or not at all.

*Dr. L. C. Leroy.*—There are two questions which I would like to ask in this connection, Is the disease infectious, and is there an hereditary tendency towards cancer?

*Dr. Dawbarn.*—I think that in cancer, as in tuberculosis, although it is not directly inherited, there may be occasionally an inherited tendency, a kind of soil in which certain seeds readily take root. One famous instance is that of the great Napoleon, who died, while still a young man, of carcinoma of the stomach. Five members of his immediate family died, I believe, of the same disease. Regarding the disease being infectious, though autoinfection is not excessively rare, there is not one well-authenticated instance where either carcinoma or sarcoma has been transmitted from the patient to the operating surgeon, although many times he has cut himself during the operation.

I would like to say in this connection that, while I have the utmost respect for Dr. Fillebrown's experience, still I do not think it would be the wise thing in a case that you believe to be sarcomatous epulis, or cancer, or any other malignant growth, to temporize with it, or to apply a caustic, hoping in this way to get rid of it. There is no doubt in my mind that in this particular case it was not true sarcoma, for had it been it would most certainly have returned, and it might then be too late to operate successfully for its thorough removal. Dr. Fillebrown presents no proof that this case of his was of a malignant nature; no pathologist examined a piece of it, which is the only true test; and hence it should not be alluded to as a cancer or a sarcoma.

*Dr. Coley.*—I have seen two or three instances myself, and have known other surgeons who have seen cases of the infectious nature of these malignant growths, at least as regards the individual. In case of sarcoma of the mesentary, for instance, after an exploratory laparotomy there was complete infection of the normal abdominal wall following the operation. A similar case of all the clean-cut surface being infected in opening up a tumor of the neck. This is one of the reasons why I prefer the Whitehead operation for cancer of the tongue.

*Dr. E. H. Babcock.*—I would like to speak of a couple of cases which I have had within the last few years, probably of epulis. One was the case of a child ten or twelve years of age in whom I observed quite a large swelling on the lower left-hand side in the molar region. This was six years ago. After scooping it out with a spoon excavator, I touched the site with chromic acid, and it has not returned.

Last fall a lady patient called, who said she had a great secret to impart to me. I examined her mouth and found on the left side, in the site of the bicuspids, a purplish swelling about the size of a hickory-nut. The upper surface was somewhat eroded but it was not pedunculated. She wanted me to handle the case, but I did not do so. I told her it would be well for her to see a surgeon at once. She went away, and I have heard nothing from her since. I felt that it was hardly wise for me to take any specimens from the growth, for fear of causing irritation and thus hastening its growth.

*Dr. Andrew J. Flanagan.*—It seems to me that the very essence of this evening's discussion was found in what Dr. Dawbarn said regarding the practitioner of dentistry having a scientific knowledge of these conditions, and I think it behooves us to prepare ourselves in this direction. At the present time it is a rare thing not to find a specialist in pathology and bacteriology in any given locality, and such being the case it is not difficult to obtain sure and scientific means to a correct diagnosis and prognosis. With the present knowledge of such cases reported here this evening, would we not be criminally negligent if we did otherwise? Dr. Brackett has referred to our beloved friend Dr. Garretson. Having been a student of his for several years, the necessity of finding the cause of a disease was well impressed on my mind. His philosophical mind well taught one thing,—never try to remove the cause until you have found it. If we do otherwise in cases such as presented here in the paper and discussion, are we not guilty of modern empiricism?

*Dr. Maxfield.*—Dr. Brackett's remarks about saying anything that will unnecessarily alarm the patient puts me in mind of the case which was ultimately sent to Dr. Coley. The patient's wife came with him each time he came to the office. At one of the later visits a dentist of my acquaintance was with me in my office. I had been speaking to him about the case, and that I thought it was a malignant tumor. At this visit, after seating the patient in the chair I called my friend, who examined the mouth and, turning to me, said, "I think as you do." I then stepped out to the patient's wife and told her the case was too serious for me to treat any longer, that it would be necessary for him to go to Boston. At that she asked me if I thought it was cancer, and began to cry. I reassured her as best I could, and endeavored to impress her with the necessity of keeping up a brave heart before her husband, and not to let him mistrust we thought it was a malignant growth. Just then I heard this gentleman say to the patient in the chair, "Yes, I think it is cancer you have got. It looks to me like a cancerous growth." That night I found the patient completely demoralized and threatening to commit suicide.

*Dr. Geo. L. Parmele.*—I wish to thank the Institute for an interesting and instructive evening. I remarked facetiously to Dr. Maxfield when I met him that I did not care for his paper, as I had already heard him talk along those lines all the way from Springfield to Boston, but I was anxious to hear all Dr. Dawbarn had to say, for I never listened to him that I did not gain valuable points. I was about to remark that I never attended but one of these meetings without getting something of value from them, and that was when I read a paper before you about one of my fads, but I remember even then I gathered some valuable information concerning my hobby in the discussion which followed my paper.

*Dr. Davenport.*—While there is really no difference in the obligation which we are placed under by the words and instruction of the three gentlemen who have entertained us to-night, we should perhaps make this distinction. Professor Dawbarn and Dr. Maxfield are our members, and we expect their brightest thoughts to be brought here. To Dr. Coley we are under especial obligations. I move you, sir, that the thanks of the Institute be extended to these three gentlemen who have entertained us to-night. Seconded and carried.

Adjourned.

FRED. L. BOGUE, M.D., D.D.S.,
*Editor The New York Institute of Stomatology.*

